# Integration of Light Signals by the Retinoblastoma Pathway in the Control of S Phase Entry in the Picophytoplanktonic Cell *Ostreococcus*

**DOI:** 10.1371/journal.pgen.1000957

**Published:** 2010-05-20

**Authors:** Mickael Moulager, Florence Corellou, Valérie Vergé, Marie-Line Escande, François-Yves Bouget

**Affiliations:** 1 Université Pierre et Marie Curie, Paris 06, Centre National de la Recherche Scientifique, Unité Mixte de Recherche 7621, Laboratoire d'Océanographie Microbienne, Observatoire Océanologique, Banyuls-sur-mer, France; 2 Centre National de la Recherche Scientifique, Unité Mixte de Recherche 7621, Université Pierre et Marie Curie, Paris 06, Laboratoire d'Océanographie Microbienne, Observatoire Océanologique, Banyuls-sur-Mer, France; 3 Université Pierre et Marie Curie, Paris 06, Observatoire Océanologique, Banyuls-sur-mer, France; University of Texas Southwestern Medical Center, Howard Hughes Medical Institute, United States of America

## Abstract

Although the decision to proceed through cell division depends largely on the metabolic status or the size of the cell, the timing of cell division is often set by internal clocks such as the circadian clock. Light is a major cue for circadian clock entrainment, and for photosynthetic organisms it is also the main source of energy supporting cell growth prior to cell division. Little is known about how light signals are integrated in the control of S phase entry. Here, we present an integrated study of light-dependent regulation of cell division in the marine green alga *Ostreococcus*. During early G1, the main genes of cell division were transcribed independently of the amount of light, and the timing of S phase did not occur prior to 6 hours after dawn. In contrast S phase commitment and the translation of a G1 A-type cyclin were dependent on the amount of light in a cAMP–dependent manner. CyclinA was shown to interact with the Retinoblastoma (Rb) protein during S phase. Down-regulating Rb bypassed the requirement for CyclinA and cAMP without altering the timing of S phase. Overexpression of CyclinA overrode the cAMP–dependent control of S phase entry and led to early cell division. Therefore, the Rb pathway appears to integrate light signals in the control of S phase entry in *Ostreococcus*, though differential transcriptional and posttranscriptional regulations of a G1 A-type cyclin. Furthermore, commitment to S phase depends on a cAMP pathway, which regulates the synthesis of CyclinA. We discuss the relative involvements of the metabolic and time/clock signals in the photoperiodic control of cell division.

## Author Summary

Microalgae from phytoplankton play an essential role in the biogeochemical cycles through carbon dioxide assimilation in the oceans where they account for more than half of organic carbon production. Photosynthetic cells use light energy for cell growth, but light can also reset the circadian clock, which is involved in the timing of cell division. How light signals are integrated in the control of cell division remains largely unknown in photosynthetic cells. We have used the marine picoeukaryotic alga *Ostreococcus* to dissect the molecular mechanisms of light-dependent control of cell division. We found that the Retinoblastoma pathway integrates light signals which regulate the synthesis of CyclinA in response to cAMP. Alteration of CyclinA or Rb levels triggers cell division in limiting light conditions and bypasses the need for cAMP. In addition, CyclinA overexpression affects the timing of S phase entry. This first integrated study of light-dependent regulation of cell division in photosynthetic cells provides insight into the underlying molecular mechanisms.

## Introduction

The cell division cycle (CDC) is a highly conserved and regulated process, which controls the proliferation of unicellular organisms and development and tissue renewal in multicellular organisms. In eukaryotes the main steps of CDC progression are controlled by Cyclin Dependent Kinases (CDKs). From human to algae, the metabolic status regulates cell cycle progression. Cell growth can occur during different phases of CDC depending on the organism but the main decision to progress into the cell cycle is usually made in G1 and depends on environmental conditions. It is referred to as cell cycle commitment and known as START in yeast or restriction point in mammals. Commitment has been depicted as a point, beyond which the cell is irreversibly engaged in cell cycle progression and is no longer sensitive to nutrients and also in the case of photosynthetic organisms, light availability [1], [2]. The transcriptional regulation of cell cycle progression in S phase is controlled in mammals and plants by the E2F transcription factors and these are sequestrated by the Retinoblastoma protein (Rb). In budding yeast, it is controlled by the transcription factor Swi4/6-dependent cell cycle box-Binding Factor (SBF) which is sequestrated by Whi5. On phosphorylation of Rb by G1 Cyclin/CDC complexes, such as CyclinD-Cdk4, E2F transcription factors are released leading to S phase commitment. In yeast on phosphorylation of Whi5, by Cln3/Cdc28, SBF is released leading to S phase commitment. In plants, a CyclinD/CDKA complex has been shown to phosphorylate a Retinoblastoma related (RBR) protein and overexpression of CyclinD accelerates entry into S phase and mitosis of G0 cells [3]. G1 cyclin/CDK complexes are primary targets of environmental signals and cyclin levels can be regulated at the transcriptional or the post-transcriptional level by mitogenic factors such as hormones and nutrients availability [4]–[7]. In animals and yeast, Rb and Whi5 respectively are critical players in linking cell size or metabolic status to cell cycle progression [8], [9].

The gating of CDC, which restricts cell division to well defined windows of time during the day, has been described for organisms as diverse as microalgae [10], [11] and mammals [12]. Gating of CDC ensures that cell division occurs with a daily periodicity over a wide range of environmental conditions. The timing of cell division is relatively insensitive to changes in the environment, such as nutrients or temperature and persists under constant light with a period close to 24 hours, two features of circadian regulation. The *Wee1* kinase, a key regulator of G2/M transition is transcriptionally regulated by the master clock complex CLOCK/BMAL1 in mouse regenerating liver cells, illustrating the direct control of cell cycle components by the circadian clock. In addition, striking experimental evidences showed that the circadian clock and the DNA damage pathway share common regulators from animals to fungi [13]–[15]. However, more experimental data is needed to unravel cross-talks between circadian, metabolic and cell cycle controls in the absence of injury or stress.

Unicellular algae such as *Chlamydomonas* or *Euglena* are very useful organisms to dissect the light-dependent regulation of cell division in photosynthetic organisms because cell division can be synchronized by light/dark cycles. In *Chlamydomonas*, commitment takes place in G1 whereas in *Euglena* the light-dependent control of CDC operates mainly in G2 but also at G1/S and S/G2 transitions [16]. In *Chlamydomonas*, cell division was shown to be under circadian control [17] but also to depend on the amount of light available for photosynthesis [1]. Until recently tools for gene function analysis were available only for *Chlamydomonas*, a microalga that exhibits multiple-fission division type. We have recently implemented molecular tools for gene function analysis in the picoeukaryotic alga *Ostreococcus tauri*, which divides by simple binary fission [18]. *O.tauri* has a very compact genome and displays very low gene redundancy [19]. A reduced set of cell cycle genes including Cyclins and Cyclin-Dependent Kinases (CDKs) were identified in the fully sequenced genome [20]. They encode functional CDKs and associated regulatory proteins [21]. Cell division and the transcription of the main cell cycle regulators were shown to be under circadian control and resetting by light demonstrated that the timing of cell division is mainly locked to the time of light on [22].

Here, we have performed an integrated study of light-dependent regulation of cell division in *Ostreococcus*, varying available light by modulating both light duration and intensity. In all conditions, the timing of cell cycle entry did not occur prior to 6 hours after dawn. No cell cycle arrest was observed outside the G1 phase. CDKA, CyclinA and Rb had patterns of expression and interactions compatible with a putative involvement in a functional Rb pathway. Cyclic AMP was necessary and sufficient for both S phase entry and CyclinA synthesis. Down-regulation of Rb or CyclinA overexpression triggered cell cycle entry under limiting light conditions demonstrating the antagonistic roles of cAMP and Rb in a “metabolic checkpoint”. Moreover, overexpression of CyclinA advanced the timing of S phase entry. Our work illustrates how combined light intensity-dependent and time-dependent signals regulate S phase entry and give insight into the role of a G1 cyclin in the light-dependent control of cell cycle progression.

## Results

### Differential effects of light intensity and duration on cell cycle commitment and timing of S phase entry

**Figure 1:**
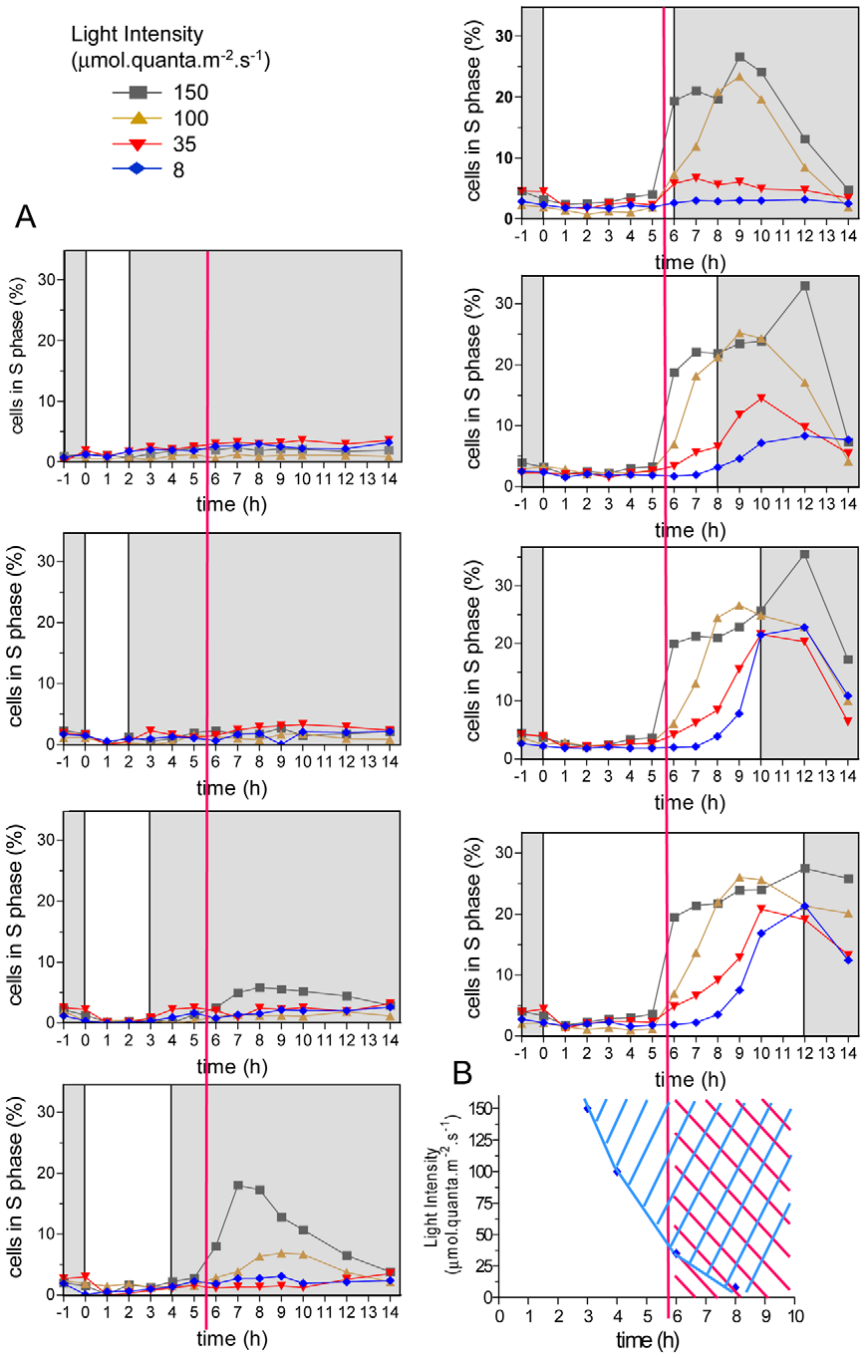
Effect of light intensity and duration of S phase on commitment and timing of S phase. (A) Cells were synchronized for five days in LD 12, 12 (same condition as the 35 µmol curve, 12 hours of light, bottom right panel) and then exposed to light of the entraining cycle of various durations (from 1 to 12 hours) and intensities (from 8 to 150 µmol quanta.m^−2^.sec^−1^) before being transferred to darkness. The percentage of cells in S phase was determined by flow cytometry (n = 20,000). For light exposure shorter than 3 hours, no cells were observed in S phase. For the highest intensity, 3 hours of light were sufficient for S phase commitment, nevertheless S phase was not observed before 6 hours after light on at all light intensities (red vertical line). At the lowest light intensity, S phase did not occur before 8 hours. (B) Earliest times of commitment are plotted at the different light intensities (blue diamond). S phase was not observed before 6 hours (red area), even when they were committed earlier under high light intensities (blue area).

Cells entrained under 12 hours light, 12 hours dark cycles (LD 12, 12) at 35 µmol.quanta. m^−2^.s^−1^ were in G1 phase at dawn (Time 0) (Figure 1). They were submitted to various light intensities and durations from Time 0 to modulate the amount of light provided (Figure 1A). Under these conditions, light is a source of energy for photosynthesis, that is required for cell growth prior to cell division (commitment) but it can also act as a signal (timer or clock) controlling the timing of cell cycle events. Estimation of DNA content by flow cytometry allowed monitoring of S phase as previously described [21], [22]. Cells in S phase were detected between 6 hours and 14 hours after light on (Time 6 and Time 14). Depending on the light intensity and duration, the cell population underwent from 0 to more than 1 division as determined by cell counting (Figure S1). G2 and M phases are very short in *Ostreococcus* as estimated from naturally or artificially synchronized cell populations [21]. Therefore the number of cells in G2/M is low and difficult to estimate [21]. Furthermore for low light intensities/durations, only a few cells divided (Figure 1) whereas for high light intensities/durations, two successive divisions could be observed (Figure S1) making it extremely difficult to estimate the rate of cells in S and G2/M phase and to discriminate between the first and second S phases. To determine the effect of light on cell division we chose to focus on the timing of entry into the first S phase (Figure 1). At the control fluence rate (35 µmol.quanta.m^−2^.s^−1^), S phase was detected from 6 hours after Light on (Time 6), that is, at the same time as under the entraining LD 12, 12 cycle. When increasing light intensity (from 35 to 100 or 150 µmol.quanta.m^−2^.s^−1^), only 3 to 4 hours of light were required for commitment to S phase. Exposure to light for 8 hours allowed S phase progression at all tested fluence rates with a maximum of cells entering S phase for highest intensities. In all conditions, cells entering S phase, completed their cell cycle and after 24 hours the cell population was back in G1. This suggests that the main light-dependent control of cell cycle progression occurs in G1 and that cell cycle progression is not impaired by darkness once cells are committed. Commitment to S phase was dependent both on light intensity and duration (blue area in Figure 1B). For example, at 150 µmol.quanta.m^−2^.s^−1^ the first committed cells were seen 2 to 3 hours before S phase was detected, whereas at 35 µmol.quanta.m^−2^.s^−1^ the first cells in S phase were detected at the same time as the first committed cells (Time 6) (Figure 1B). For the lowest light intensity, the timing of S phase was delayed (red area in Figure 1B), most likely because cells had not received enough light to commit at that time (intersection of the blue and red area on Figure 1B). Together these results indicate that the timing of entry into the first S phase is gated during several hours after dawn. For the highest light intensities some cells were able to divide twice in a row (Figure S1), suggesting that the timing mechanism which gates cell division until Time 5 to Time 6, has little effect on the timing of the second division. This is similar to the gating of division described in *Chlamydomonas*, which is restricted to a time window, the number of successive divisions being determined by the light conditions [1].

### Light-dependent regulation of cell division cycle genes transcription

**Figure 2:**
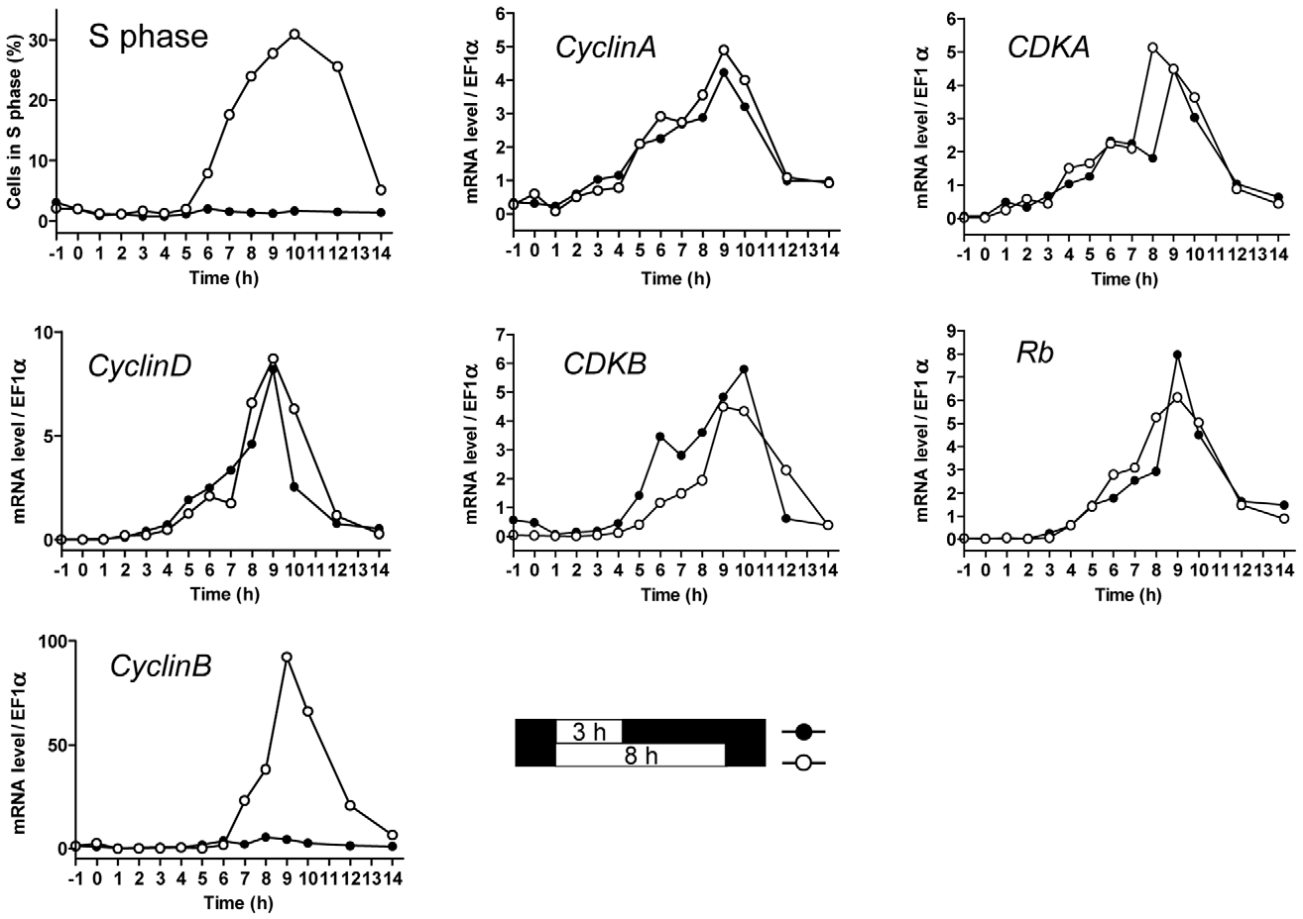
Transcription of G1 regulators occurs independently of commitment. Cells were grown in LD 12, 12 at 35 µmol quanta.m^−2^.sec^−1^ for 5 days and then exposed to light of 100 µmol quanta.m^−2^.sec^−1^ for 3 hours (limiting conditions, filled circles) in which no cell entered in S phase, or 8 hours in which the population doubled (open circles) as shown in Figure 2. The proportion of cells in S phase was monitored by flow cytometry. Relative expression levels of *CyclinA*, *CDKA*, *CyclinD*, *CDKB*, *Retinoblastoma* and *CyclinB* were monitored using real time quantitative RT-PCR. Relative expression level is normalized to the housekeeping gene *EFα*. Values are mean of duplicates. Note that except for the mitotic *CyclinB*, which was expressed only in non-limiting conditions, all other cell cycle genes had similar transcription profiles for both light conditions.

Limiting and non-limiting light conditions for cell division (referred to as limiting and non-limiting conditions) were chosen as three and eight hours respectively, of exposure to light at 100 µmol.quanta.m^−2^.s^−1^. At this level about 90% of an LD 12, 12 entrained cell population divided (see Figure S1). We investigated the transcription patterns of the main cell cycle actors of cell division in limiting and non-limiting conditions (Figure 2). Transcription of *Cyclin-Dependent Kinases* (*CDKs*), *Cyclins* and *Retinoblastoma* (*Rb*) were monitored by quantitative RT-PCR. *CyclinB* was the only transcript that was not detected in limiting conditions, whereas expression patterns of other cell cycle genes including *CyclinA*, *CyclinD*, *CDKA*, *CDKB* and *Rb* remained similar in both limiting and non-limiting conditions. *CDKA* and *CyclinA* transcripts were detected first, accumulating as early as two hours after light on, closely followed by *Rb*, *CyclinD* and *CDKB* mRNAs. Maximal transcripts levels were observed between 9 to 10 hours after light on when most of the cells were progressing through the cell cycle.

In the non-limiting light condition the transcription of *CyclinB* started after 6 hours, when S phase had begun, suggesting that its transcription might be dependent on cell cycle progression in G1. In contrast known and putative G1/S regulators, including *CDKA*, *CyclinA* and *Rb*, were not differentially expressed in limiting and non-limiting conditions, indicating that their transcriptional regulation is independent of commitment.

### CyclinA interacts with both the Retinoblastoma protein and CDKA and is differentially expressed under various light conditions

**Figure 3:**
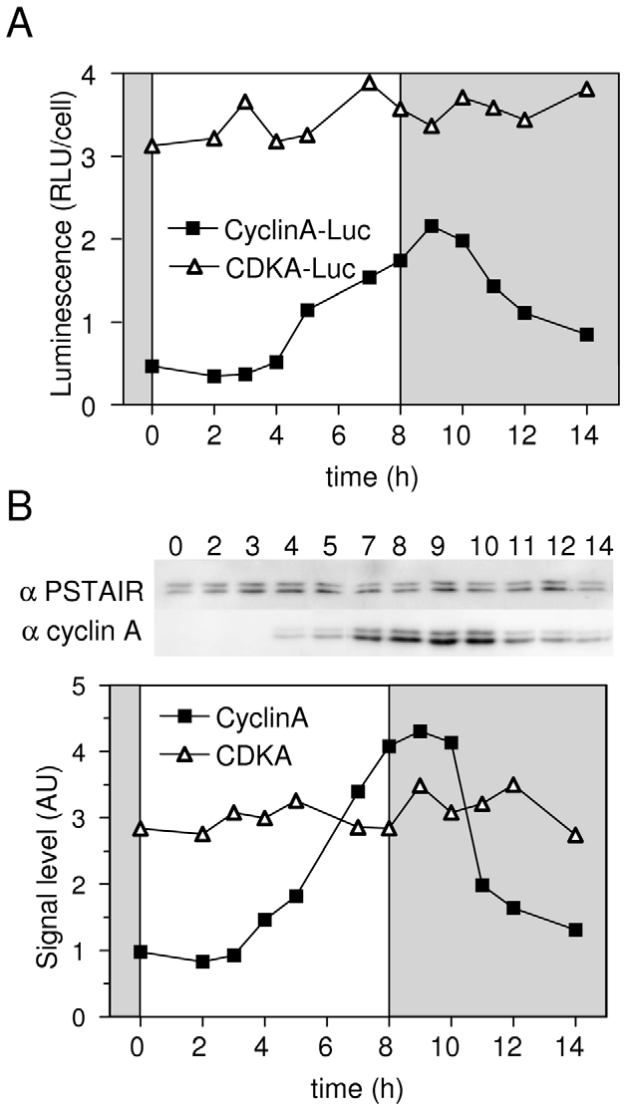
Validation of luciferase translational reporters to estimate CyclinA and CDKA levels in protein extracts. CyclinA-Luc and CDKA-Luc lines and wild type cells (WT) entrained in LD 12, 12 were subjected to 8 hours of light at 100 µmol quanta.m^−2^.sec^−1^ from dawn. The level of proteins was determined by luminescence of luciferase translational reporters (A) or by western blot on wild type cells using anti-CyclinA antibody or anti-PSTAIR antibody for CDKA (B). The patterns of luminescence of translation reporters reflect the profiles of expression of endogenous proteins as determined by Western Blot analysis. RLU: Relative luminescence unit. AU: arbitrary unit.

Together Figure 1 and Figure 2 suggested that commitment occurs upon light assimilation in G1 and that it does not primarily rely on transcriptional regulations of the putative G1/S regulators identified *in silico*. Because, the Retinoblastoma protein (Rb) is well known to play a central role in the restriction point of plant and animal cells, we chose to investigate the role of Rb in G1 progression in *Ostreococcus*. CyclinA is the only protein exhibiting a canonical (LXCXE) Rb-binding site [23] and CDKA is the only CDK expressed in G1. Thus, CDKA/CyclinA complex is the best candidate for regulating cell cycle progression in G1. To monitor Rb, CDKA, and CyclinA protein synthesis and quantify interacting partners, we generated stable translational luciferase reporter lines Rb-Luc, CDKA-Luc and CyclinA-Luc in the pOtLuc vector [18]. Estimation of the recombinant protein synthesis was achieved through luminescence measurement from either whole protein extracts or affinity-purified proteins. The human p9^CKShs1^ referred to as P9 was used to specifically purify CDKA [21]. An anti-CyclinA antibody was used for immunoprecipitation of CyclinA and associated proteins. Luminescence patterns measured in extracts from CDKA-Luc and CyclinA-Luc lines were similar to that of CDKA and CyclinA profiles as determined by western blot, demonstrating that in our experiments luciferase translational fusions (Figure 3A) reflected the expression patterns of these proteins (Figure 3B), which in the case of CyclinA resulted mainly from protein *de novo* synthesis since endogenous CyclinA was no detected at Time 0 (Figure 3A and 3B).

**Figure 4:**
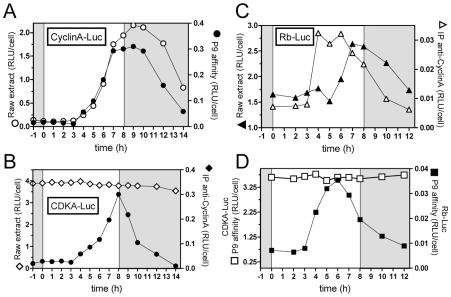
Interactions among CyclinA, CDKA, and Rb proteins under non-limiting light conditions. Luminescence assays of Luciferase fusion proteins were performed either on raw extract or after affinity precipitation of proteins bound to CDKA using the P9 protein or to CyclinA using a specific antibody. (A) CyclinA-Luc translational reporter lines was grown in LD 12, 12 at 35 µmol quanta.m^−2^.sec^−1^ for 5 days and then exposed to light of 100 µmol quanta.m^−2^.sec^−1^ for 8 hours (non-limiting conditions). Luminescence of CyclinA-Luc in raw extracts (open circles) had a similar profile as CyclinA-Luc associated to CDKA, purified on P9 (filled circles). (B) CDKA-Luc displayed a steady state level in raw extracts (open diamonds). CDKA-Luc bound to CyclinA immunopurified using a specific antibody to CyclinA peaked 8 hours after light on (filled diamonds). (C) Rb-Luc level peaked 8 hours after light on (filled triangles). Rb-Luc was found maximally bound to CyclinA 3 hours before (open triangles), suggesting that CyclinA/Rb interactions is not correlated to the amount of Rb. (D) While CDKA-Luc bound to P9 showed constant levels (open squares), Rb-Luc was found associated to CDKA 5 to 6 hours after light on (filled squares), that is several hours before CyclinA/CDKA maximal interaction. RLU: relative luminescence unit on the Y axis. Experiments were performed in duplicate. Each graph shows a representative experiment.

In non-limiting conditions, CyclinA-Luc accumulated from 4 hours after light on (Figure 4A). Similar profiles of CyclinA-Luc were obtained from raw extracts or P9-purified complexes (CDKA/CyclinA-Luc) (Figure 4A). Significantly, CyclinA-Luc protein was found to be bound to CDKA from Time 4 that is, as soon as CyclinA-Luc was detected in raw extracts. Conversely, CyclinA/CDKA-Luc complexes were immuno-precipitated with the anti-CyclinA antibody. While a steady state level of CDKA-Luc was detected in raw extract, the amount of CDKA-Luc copurified with CyclinA followed the profile of CyclinA-Luc in raw extract (Figure 4B). These results suggest that CyclinA may be a limiting factor in the formation of the CyclinA/CDKA complex, before commitment.

Rb-Luc level increased from 6 hours after light on, reaching a maximum at 7 to 8 hours after light on (Figure 4C). In contrast, Rb-Luc bound to CyclinA in immunoprecipitation experiments peaked 3 hours before Rb-Luc in raw extract (Figure 4C). Rb-Luc purified on P9 (bound to CDKA) had a similar profile as Rb-Luc bound to CyclinA (Figure 4D and 4C). Since CDKA-Luc was detected at a steady state level in raw extracts (Figure 4B), this suggests that CyclinA might be a limiting factor in CDKA/Rb interaction early after dawn. Significantly, the amount of Rb-Luc associated to CyclinA or CDKA was highest around Time 5 to Time 6, that is, 2 hours before CDKA/CyclinA maximal interaction. This suggests that at the light/dark transition (Time 8), Rb is released from the CyclinA/CDKA complex.

**Figure 5:**
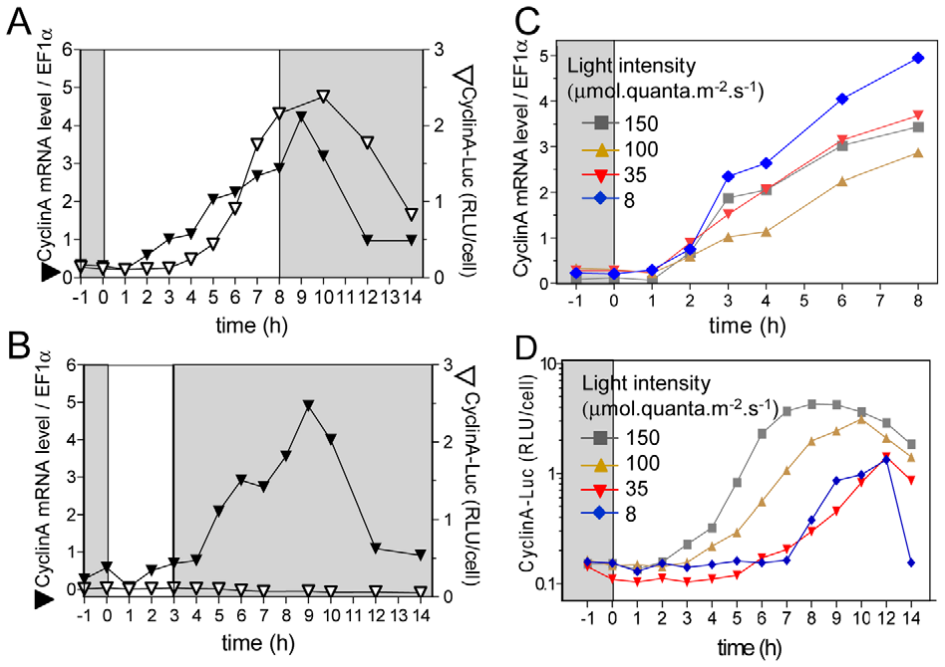
Differential regulation of CyclinA transcription and protein accumulation during commitment. Cells were grown in LD 12, 12 at 35 µmol quanta.m^−2^.sec^−1^ for 5 days and then exposed to light of 100 µmol quanta.m^−2^.sec^−1^ for 8 hours (A) or 3 hours (B). *CyclinA* mRNA levels were monitored by real time RT-PCR (filled inverted triangles) and CyclinA-Luc protein accumulation by luciferase assay (open inverted triangle). (C) and (D) Cells were grown in LD 12, 12 at 35 µmol quanta.m^−2^.sec^−1^ for 5 days and then exposed to 12 hours of light at various intensities from 8 to 150 µmol quanta.m^−2^.sec^−1^ as described in Figure 1 (corresponding colour codes). *CyclinA* transcription profiles were similar in all conditions (C). In contrast CyclinA-Luc protein was detected later when lowering light intensity (D). RLU: relative luminescence unit on the Y axis.

*CyclinA* transcript and CyclinA-Luc were monitored in limiting and non-limiting conditions (Figure 5). In non-limiting conditions CyclinA-Luc was detected from 4 hours after light on, i.e. two hours after *CyclinA* transcript (Figure 5A). No CyclinA-Luc could be detected in limiting conditions though *CyclinA* mRNA profile remained similar to that in non-limiting conditions (Figure 5B). When the light supply was modulated by changing the fluence rate instead of the duration of illumination (12 hours of light), all profiles of *CyclinA* mRNA were increasing from 1 hour after light on (Figure 5C). In contrast, translation products, as reported by CyclinA-Luc, appeared later for lower fluence rates (Figure 5D). These results indicate that the synthesis of CyclinA protein, unlike the *CyclinA* transcription, is regulated by the light conditions in a manner very similar to S phase commitment (e.g. Figure 1).

### Cyclic AMP regulates CyclinA protein level and cell division

To gain insight into the signal transduction pathway leading to the light-dependent regulation of S phase entry, we chose to investigate the involvement of cAMP known to be an important signaling component for cell cycle progression [24]–[26]. Monitoring of cAMP level in cells exposed to various light intensities after LD 12, 12 entrainment revealed that the peak of cAMP occurred earlier and/or had higher amplitudes for high fluence rates (Figure S2). This suggests a possible correlation between cAMP level, CyclinA synthesis and S phase commitment since cells were committed sooner at high fluence rates (e.g. Figure 1).

**Figure 6:**
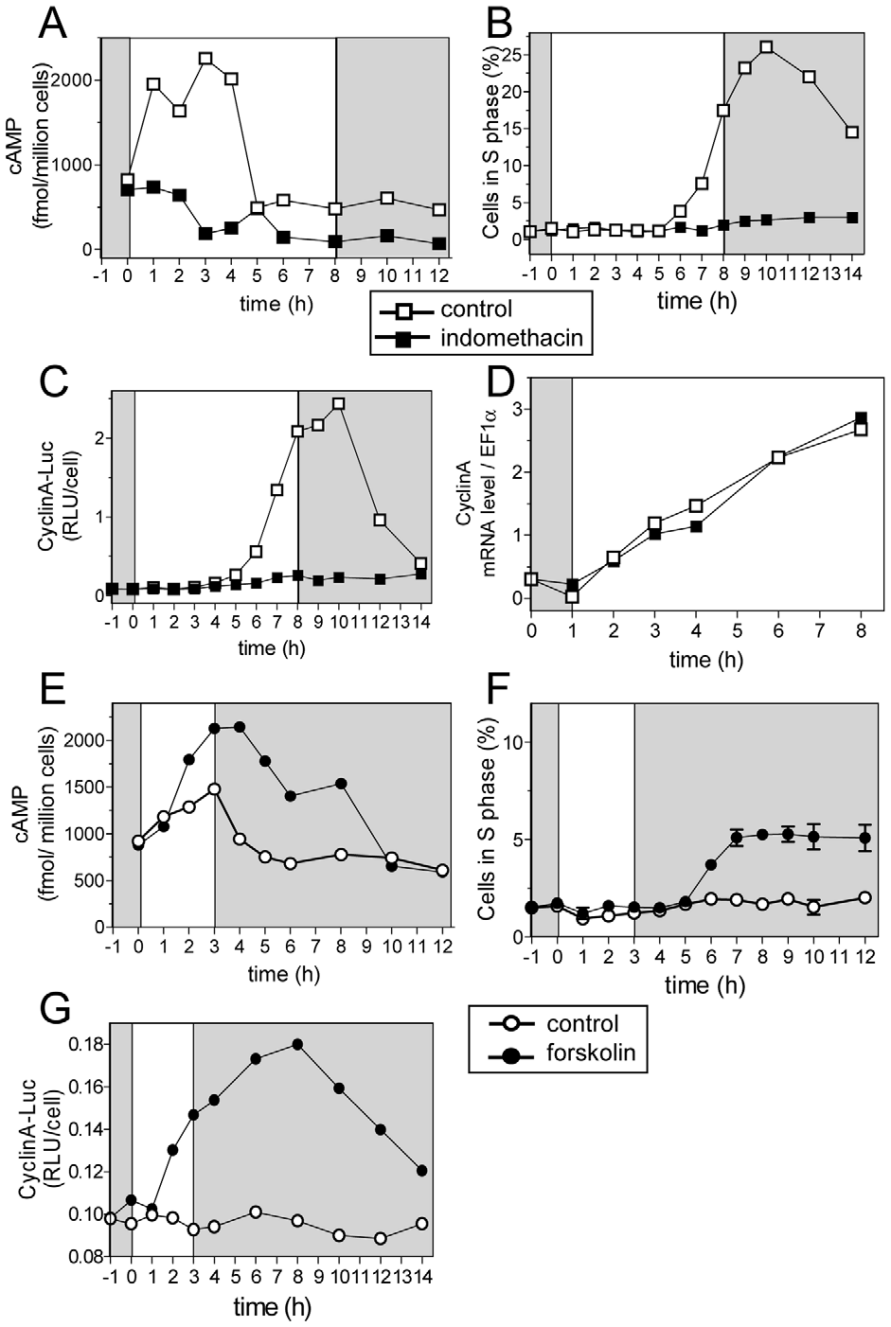
Cyclic AMP regulates S phase entry and CyclinA synthesis. Cells were entrained in LD 12, 12 at 35 µmol quanta.m^−2^.sec^−1^ for 5 days and then exposed to light of 100 µmol quanta.m^−2^.sec^−1^ for 8 hours (open squares). (A–D). Indomethacin (30 µM) was added at Time 1 to inhibit cAMP synthesis (filled squares). (A) cAMP levels. (B) S phase. (C) CyclinA-Luc protein accumulation. (D). CyclinA mRNA relative to EF1α as determined by real time RT-PCR. (E–G) Forskolin (20 µM) was added at Time 0 to trigger cAMP synthesis. (E) cAMP levels. (F) S phase. A low percentage of cells in S phase was detected but it was significantly above control. Error bars represent SD. (G) CyclinA-Luc protein.

Under non-limiting conditions, cAMP increased immediately after light on and returned to a basal level before S phase was detected (Figure 6A and 6B). We used a pharmacological approach to evaluate the role of cAMP in the synthesis of CyclinA and the control of S phase. Indomethacin and forskolin have been reported to inhibit and activate cAMP synthesis, respectively, including in photosynthetic organisms [27], [28]. Inhibiting cAMP synthesis with indomethacin (30 µM) impaired cell cycle entry and CyclinA-Luc protein synthesis without affecting *CyclinA* transcription (Figure 6A–6D). Conversely, the adenylate cyclase activator forskolin (20 µM) significantly increased cAMP levels under limiting light conditions (Figure 6E). Remarkably, S phase cells as well as CyclinA-Luc protein synthesis were detected, though at a low levels, in the presence of forskolin (Figure 6F and 6G). CyclinA-Luc accumulated as early as one hour after forskolin addition, i.e. two hours after light on but the first cells in were not detected before 6 hours after light on as in control cells. Our results indicate that cAMP is required for CyclinA synthesis and S phase entry. The delay between the peak of cAMP and commitment suggests that cAMP is an upstream signal in a signal transduction pathway leading ultimately to commitment rather than a direct regulator of commitment.

### Ectopic expression of CyclinA triggers early S phase entry under limiting light conditions and when cAMP synthesis is inhibited

**Figure 7:**
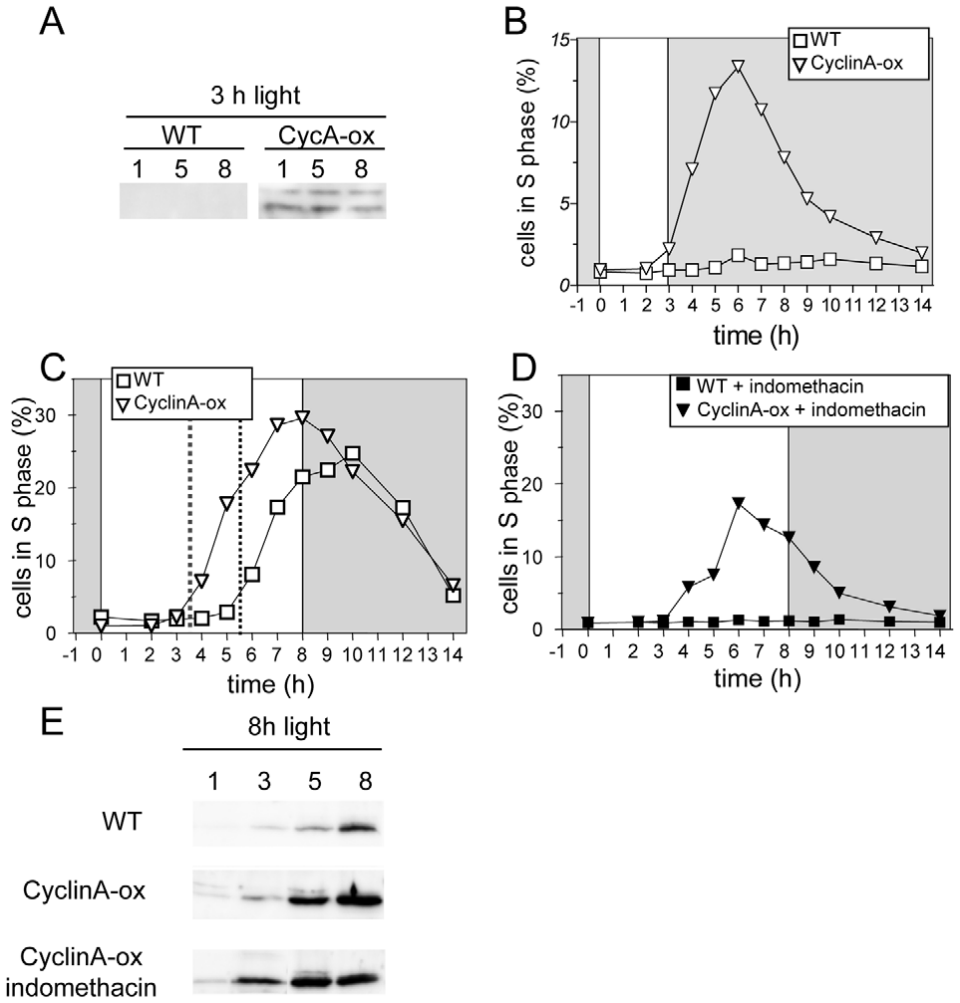
CyclinA overexpression induces S phase entry with an earlier timing in limiting conditions. Wild-type cells (WT, squares) and CyclinA-ox cells (inverted triangles) were entrained in LD 12, 12 for 5 days and then exposed to light of 100 µmol quanta.m^−2^.sec^−1^ for 3 hours (A,B) or 8 hours (C–E). (A) Western Blot analysis of CyclinA levels in CyclinA-ox and WT in limiting light conditions (3 h light). (B) S phase in CyclinA-ox (open inverted triangles) and WT (open squares) under limiting light conditions. (C) Under non-limiting conditions, CyclinA-ox line (open inverted triangles) entered S phase 2 hours before WT (open squares) as pointed by the red dotted line. (D) Addition of indomethacin (30 µM) one hour after dawn prevented S phase entry in WT cells (filled squares) but S phase was still observed in CyclinA-ox line (filled inverted triangles). (E) Western Blot analysis of CyclinA levels in WT, CyclinA-ox and indomethacin-treated CyclinA-ox in non-limiting light conditions (8 h light).

To better understand the respective involvement of CyclinA protein and cAMP in commitment, CyclinA was ectopically expressed under the strong and constitutive *Ostreococcus* High Affinity Phosphate Transporter (HAPT) promoter in the pOtoxLuc vector (Figure S3). Two lines, referred to as CyclinA-ox, were selected based on CyclinA expression levels. In limiting conditions CyclinA was detectable, though at low levels, as early as 1 hour after light on in CyclinA-ox lines (Figure 7A). In CyclinA-ox line S phase was detected under limiting light conditions as early as 3 hours after light on (Figure 7B). Under non-limiting conditions S phase entry was advanced by two hours in CyclinA-ox lines compared to WT cells (Figure 7C). Moreover, unlike control cells, CyclinA–ox cells treated with indomethacin were able to enter S phase (Figure 7D). Under non-limiting conditions, CyclinA displayed higher levels than control cells at all time points (Figure 7E). Noteworthy, the levels of CyclinA increased after dawn in CyclinA-ox lines as in control cells, suggesting that post- translational regulations operate also in overexpression lines. Higher levels of CyclinA were also detected in indomethacin-treated cells (Figure 7E).

Overexpression of CyclinA, therefore, bypasses the requirement for light and cAMP, allowing cells to commit and to enter into S phase with an earlier timing.

### Down-regulation of the Retinoblastoma protein synthesis allows S phase entry in limiting light conditions

**Figure 8:**
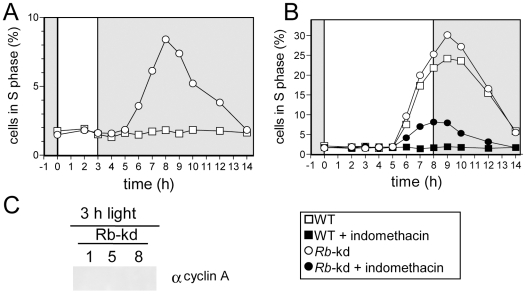
Retinoblastoma knock-down triggers S phase entry in limiting light conditions but has no effect on the timing of cell division. Wild-type cells (WT, squares) and Rb-kd cells (circles) were entrained in LD 12, 12 for 5 days and then exposed to light of 100 µmol quanta.m^−2^.sec^−1^ for 3 hours (A,C) or 8 hours (B). (A) Cell in S phase in Rb-kd and WT cells under limiting conditions. (B) Under non-limiting conditions, Rb-kd cells (open circles) entered S phase at the same time as WT cells (open squares). Addition of indomethacin (30 µM) one hour after light on, prevented S phase entry in WT cells (filled squares) but S phase was still detected in Rb-kd cells (filled circles). (C) Western blot analysis of CyclinA levels in Rb-kd line in limiting conditions (3 h light) 1, 5 and 8 hours after light on. No CyclinA was detected unlike in control WT cells grown under non-limiting conditions (Figure 7A). Note that these samples were processed and resolved on the same gel as in Figure 7A, which can be considered as a positive control.

To test the role of Rb in the light-dependent regulation of cell division, we generated Retinoblastoma- knockdown (Rb-kd) by expressing antisense Rb sequence in the pOtoxLuc vector. Three lines, with reduced levels of *Rb* mRNA compared to WT cells were selected (Figure S4). Under limiting conditions, Rb-kd cells were still able to progress into S phase (Figure 8A). Inhibition of cAMP synthesis by indomethacin reduced the number of Rb-kd that entered S phase but a significant proportion progressed through S phase, while wild type cells remained in G1. Thus, down-regulation of Rb bypasses the need for light and cAMP in Rb-kd as in CyclinA-ox lines. Entry into S phase occurred at the same time in Rb-kd cells as in WT under non-limiting light conditions (Figure 8C). Therefore repression of Rb expression triggers commitment but has no obvious effect on the timing of S phase, unlike overexpression of CyclinA, which induces an earlier timing of cell division. Significantly, in limiting light conditions CyclinA was not detected in Rb*-*kd cells (Figure 8C) as in control cells (e.g. Figure 7A). As cells in S phase were observed in Rb-kd cells, this suggests that CyclinA may not be essential for S phase entry when Rb is repressed in limiting light conditions.

## Discussion

### Light-dependent control of cell division occurs in G1 phase

We have previously shown that in *Ostreococcus*, the CDC is synchronized by day/night cycles and that a timing mechanism, namely the circadian clock, regulates cell division and the transcription of the main cell cycle regulators [22]. The present study aims to decipher the molecular mechanisms involved in the photoperiodic control of cell division. Varying the fluence rate and/or the duration of exposure to light modulates the length of the G1 phase. Once committed, cells resume division indicating that the light-dependent regulation of cell division occurs mainly in G1 (Figure 1). G1 phase is lengthened by as much as 3 hours (from 6 to 9 hours) for the lowest light intensity, suggesting that metabolic status might control commitment. Conversely, for high fluence rates, 3 to 4 hours are sufficient for commitment but S phase is not observed prior to 6 hours after dawn indicating that engagement of committed cells into S phase is gated in time. Such a light-dependent control of cell cycle progression has been described in *Chlamydomonas*. In early G1, cell cycle progression is light-dependent but after commitment it becomes light-independent and cells wait for an additional 5 to 10 hours before entering S phase [1]. In contrast, in *Euglena*, the light-dependent regulation of cell division occurs in both G1 and G2 phases because on transfer to darkness cell cycle arrested at both stages [16].

### Commitment is controlled by the retinoblastoma pathway

**Figure 9:**
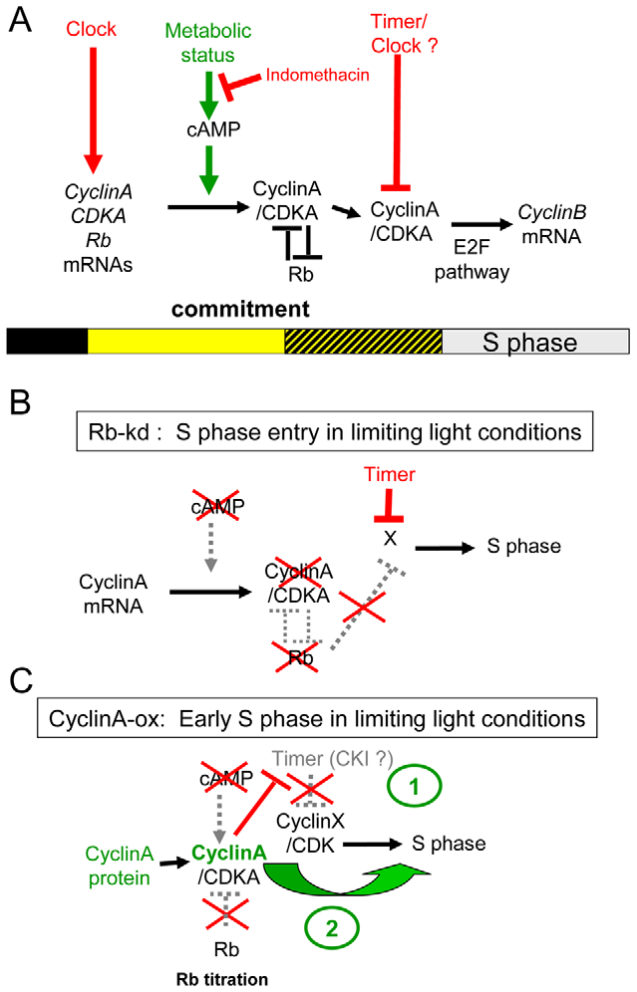
Speculative model of the light-dependent regulation of S phase entry in *Ostreococcus*. (A) In WT cells, the transcription of the main cell cycle regulators is under circadian control. Commitment to S phase depends on both the light intensity and duration from dawn (light represented by yellow square). Therefore the precommitment period varies in length (dashed square). In contrast, S phase is gated by a timer and is not detected before 6 hours after dawn. During precommitment CyclinA is synthesized under control of a cAMP dependent pathway. The cyclinA/CDKA complex promotes entry into S phase in committed cells. (B) Rb-knockdown cells are able to enter S phase in limiting conditions or when cAMP synthesis is inhibited. In these lines, CyclinA is not detected under limiting light conditions suggesting that CyclinA may be required to overcome the inhibitory effect of Rb in WT cells. However, the gating of S phase by a timer still operates in Rb-kd cells. (C) Under limiting conditions CyclinA-ox cells commit to S phase even when cAMP synthesis is inhibited. CyclinA-ox cells enter into S phase earlier than WT or Rb kd cells. Overexpression of CyclinA may non-specifically induce cell cycle progression by functionally replacing another cyclin (CyclinX) (1) and/or titer an inhibitor such as CDK Inhibitor (CKI) which would gate S phase in WT cells (2).

The main cell cycle regulatory genes display similar patterns of transcription under limiting and non-limiting light conditions (Figure 2). From completion of mitosis (about 2 to 3 hours after light off) to the early morning, no transcription of the main cell cycle regulators is detected [22]. *CyclinA* is the first gene to be transcribed from one hour after light on closely followed by *CDKA*, *CyclinD*, *CDKB* and *Rb*. (Figure 9). Transcription of these genes occurs at fixed time intervals from light on independently of the commitment status of the cells, suggesting that transcription is mainly controlled by a dawn-dependent timing mechanism, which would be similar to the circadian control of cell division [22]. The main regulatory genes of cell division, including cyclins and CDKs have been shown to retain rhythmic expression under constant light [22] suggesting a circadian regulation of their transcription. We show here that *CyclinB* transcription is further dependent on commitment (Figure 2). The presence of an E2F-binding motif in the promoter of *CyclinB*, suggests that *CyclinB* transcription may depend on E2F transcription factor, once the cells have passed commitment. Besides being the first cyclin to be expressed soon after dawn, CyclinA is the only cyclin, which displays a fully conserved Rb-interaction motif in *O. tauri*. Furthermore CyclinA interacts with both CDKA and Rb protein in G1 (Figure 4). Since CDKA is the only canonical CDK present during G1 phase [21], CDKA/CyclinA, is potentially the only CDK/cyclin complex in the Rb pathway in *Ostreococcus*. As soon as CyclinA protein is detected, it is found in association with both CDKA and Rb protein. In contrast, CDKA does not associate with Rb in the absence of CyclinA suggesting that CyclinA may be essential for the formation of this complex. Finally CyclinA remains complexed with CDKA several hours after the maximal interaction between Rb/CyclinA and Rb/CDKA, consistent with the CyclinA/CDKA complex being involved in the control of S phase entry. Surprisingly, the level of Rb remains high during S phase, which may appear to be in contradiction with an exclusive role for Rb in S phase progression. This suggests that Rb might also be involved later during cell cycle progression as previously reported in several organisms [29]–[31].

CyclinA overexpression or Rb down-regulation induce cell division in limiting light conditions indicating that the Retinoblastoma pathway plays an essential role in cell cycle progression at commitment (Figure 9B and 9C). Such a commitment phenotype has been observed in animal knockout cells lacking the entire *Retinoblastoma* family, which cannot undergo growth arrest when starved of growth factors [32] and *Chlamydomonas mat3* mutants cells were shown to divide at smaller size than wild type cells [33]. The best known function of Rb is to repress S phase transcription by sequestering the E2F transcription factor until Rb is phosphorylated by specific Cyclin/CDK complexes [34]. Our results would be consistent with CyclinA/CDKA controlling cell cycle progression in G1 by regulating Rb phosphorylation at commitment, Rb being a negative regulator of CyclinA/CDKA (Figure 9A). No CyclinA was detected in Rb-kd cells entering early into S phase under limiting light conditions. This would suggest that other cell cycle regulators downstream of CyclinA that are not under the control of Rb are sufficient to promote S phase entry in the absence of CyclinA (Figure 9B). Therefore a main function of CyclinA/CDKA may be to override the inhibitory effect of Rb in wild type cells (Figure 9A).

### Cyclic AMP is a secondary messenger necessary for commitment and CyclinA synthesis

In non-limiting light conditions, the level of cAMP increases from light on and peaks before S phase entry (Figure S2), while cAMP level remains low under limiting light conditions (Figure 6). Mitogens such as the EGF are known to induce cell cycle re-entry in a cAMP dependent manner [35]. In *S. cerevisiae*, G1 progression is regulated by cAMP, which mediates the intracellular level of glucose [26]. In *Ostrecoccus* the inhibition of cAMP synthesis with indomethacin prevents cAMP accumulation and cell division under non-limiting conditions but has no effect on cell growth (Figure 6). Conversely forskolin, an activator of cAMP synthesis, triggers cell division, though at low rates, under limiting light conditions. The fact that cAMP is necessary for commitment suggests that our limiting light condition may correspond to a metabolic limitation by restricting the light energy available for photosynthesis (Figure 9A).

The transcription of *CyclinA* does not depend on light conditions but the synthesis of CyclinA protein occurs only under non-limiting light conditions. When CyclinA protein is detected earlier under high light, the cells commit sooner. While the rise in transcription of *CyclinA* is independent of the light conditions occurring at a fixed time after light on, the post-transcriptional regulation of CyclinA may ensure that CyclinA does not accumulate until optimal metabolic conditions allowing cell growth for commitment are met (Figure 9A). Inhibition of cAMP synthesis prevents CyclinA synthesis under non-limiting light conditions and activation of cAMP synthesis triggers CyclinA synthesis consistent with CyclinA accumulation being regulated by cAMP levels. In agreement with this hypothesis, down-regulation of Rb or overexpression of CyclinA bypasses the need for cAMP for cell division (Figure 9B and 9C). A similar mechanism of G1 progression by the metabolic status has been described in yeast. The differential translation rate of the G1 cyclin cln3 is dependent on a Ras-cAMP pathway, which reflects the metabolic status of the cell [26]. In a rich carbon source, cln3 appears earlier, interacts with cdc28 to phosphorylate Whi5 and induces an earlier progression through START, leading to a shortening of G1 phase. Our results suggest that in *Ostreococcus* cells the synthesis of CyclinA is regulated by a cAMP dependent mechanism when cells have accumulated enough light energy (Figure 9A). However, the time lapse between the peak of cAMP and S phase observed in most of the light conditions suggests that cAMP does not directly control CyclinA synthesis but that it activates a downstream pathway that controls CyclinA accumulation. An alternative explanation is that cAMP regulates cell cycle progression independently of commitment, consistent with the fact that the temporal changes in cAMP level do not correlate exactly with the timing of the commitment.

### What is the timing mechanism gating S phase entry?

The timing of cell division in *Ostreococcus* has been shown previously to be regulated mainly by the dark-light transition and to occur in G1 because perturbations by light or dark pulses induced changes in timing of S phase entry, but not in the duration of the S, G2 and M phases [22]. In Figure 1, we show that the timing of S phase is delayed when entrained cells are exposed to low light from dawn. In all conditions S phase is not observed before 6 hours after light on, even for high fluence rates suggesting that the timing of S phase is gated (Figure 1). Previous light-resetting experiments have shown that the timing of cell division is mainly locked to light on at dawn defining a time window in which cells do not divide [22]. Timing mechanisms of cell division have been shown to rely on clocks such as the circadian clock in animal cells, which regulates cell cycle progression during both G1 and G2 phase. [36]–[38]. In mice liver cells re-entering the cell cycle upon liver partial ablation, the circadian clock gates entry into mitosis by regulating the transcription of the Wee1 kinase, which inhibits the activity of Cyclin B1/CDC2 kinase in G2 [36]. In *Ostreococcus* cells, rhythmic patterns of cell division and transcription of the main cell cycle regulators persist under constant light, supporting a circadian regulation of cell division [22]. *CyclinA* transcription is not affected under a wide range of light conditions further suggesting that it is regulated by a timing mechanism rather than by metabolic control. This would be consistent with a clock controlling the transcription of the main cell cycle actors after dawn. Alternatively we cannot rule out the possibility that the light on signal, on its own, is sufficient to trigger the transcription of the main cell cycle regulators in G1.

Remarkably, overexpression of CyclinA induces earlier entry into S phase in limiting light conditions, suggesting at first sight that the regulation of CDKA/CyclinA activity by a clock may account for the timing of S phase (Figure 7). However Rb-kd cells enter S phase without any detectable CyclinA under limiting light conditions and in these cells the timing of S phase is normal (Figure 8 and Figure 9B). As mentioned above it is possible that CyclinA/CDKA promotes S phase entry by counteracting the inhibitory effect of Rb (Figure 9A). In the absence of CyclinA and Rb, S phase would be controlled by another timing mechanism. An as yet unknown player, such as a Cyclin/CDK complex would control S phase entry independently of CyclinA/CDKA and it would be negatively regulated by Rb in normal conditions. Our results also suggest that this player would be controlled by an independent timing mechanism since the timing of S phase is normal in Rb-kd lines which display low levels of Rb transcript and no detectable CyclinA protein under limiting light conditions (Figure 9B). The earlier timing of S phase entry observed in CyclinA-ox lines under limiting light conditions could also be explained by a titration effect of an inhibitor such as a CDK inhibitor by CyclinA (Figure 9C). It is also possible that CyclinA overexpression non-specifically induces cell cycle progression by replacing another Cyclin/CDK complex since cyclins can have overlapping functions. Alternatively, the overexpression of CyclinA may induce cell cycle progression downstream of the G1/S progression if CyclinA is also involved later during cell cycle progression as previously hypothesized [21].

Finally, activation of cAMP synthesis by forskolin induces an early synthesis of CyclinA though at low levels but the timing of S phase is not advanced. It is therefore possible that CyclinA promotes cell cycle progression in a dose-dependent manner and that the forskolin-treatment prevents CyclinA from reaching sufficient levels to promote early S phase entry.

In summary, we propose a model of the light-dependent regulation of G1 phase progression, in which timing and metabolic signals are integrated in a sequential way (Figure 9A). First, the transcription of several genes needed for G1 phase progression is activated, independently of the amount of light provided. Among these genes, *CyclinA* is one of the earliest to be transcribed after dawn, likely depending on a timing mechanism such as the circadian clock. CyclinA synthesis appears to be controlled by a cAMP-dependent pathway, most probably under metabolic control. CyclinA protein binds both CDKA and Rb, which might result eventually in the release of E2F transcription factor upon phosphorylation of Rb. Finally another timing mechanism, independent of commitment prevents entry into S phase before 6 hours after dawn. Whether this timer is the circadian clock and which cell cycle regulators are involved is currently unknown. The limited number of cell cycle regulators in *Ostreococcus* as well as the recent identification of circadian clock players [18] should allow this question to be addressed in the future.

More generally, unraveling the molecular mechanisms of light-dependent regulation of growth and cell division in microalgae from the phytoplankton should lead to a better understanding of the physiology of these key organisms involved in carbon dioxide assimilation.

## Materials and Methods

### Algal materials, culture and harvesting conditions

*Ostreococcus* tauri strain, 0TTH0595 isolated from the Thau lagoon [39], was cultivated in filtered sterile seawater supplemented with Keller enrichment medium (Sigma-Aldrich, Lyon, France). *O. tauri* strain was grown in aerated flasks (Sarstedt) at 20°C under 12 hours light/12 hours dark cycles as previously described [21]. Drugs were purchased from Sigma-Aldrich unless otherwise stated. For extraction, cells were harvested by centrifugation in conical bottles (10,000 g, 10 min, 4°C), after addition of pluronic (0.1%) to the medium and stored at −80°C until extraction. Frozen cell-pellets were ground by shaking (45 seconds, 30 Hz, twice) with 5 mm stainless steel beads using a TissueLyser (Retsch, Haan, Germany) after the appropriate buffer was added. Cell debris were removed by centrifugation (12 000 g, 10 min, 4°C).

### Flow cytometry analysis, cell cycle statistic analysis

A 1 ml cell sample was fixed with 0.25% glutaraldehyde (Sigma) for 15 min at room temperature and then stored at 4°C for 1 day or frozen in liquid nitrogen and stored at −80°C. Flow cytometry analysis was performed on a FACScan flow cytometer (FACScalibur; Becton-Dickinson, San Jose, CA). Cells were counted from the appropriate gate (FL3-H versus SSC-H) as described previously [39]. For analysis of the DNA content, whole fixed cells were stained with SYBR green I (3000X dilution of the commercial solution; Molecular Probes, Eugene, OR) for 30 min, and 20,000 cells per sample were analyzed using the CellQuest software. Cell cycle analysis was performed with the Modfit software (Verity Software House, Tophsam, ME) as previously described [21]. The Graphical trapezoidal model and the fixed ratio of G2/G1 of 1.85 gave the best fits and were kept for cell cycle analysis in all analysis.

### Cloning strategy and vectors

Amplifications by PCR of *CDKA*, *CyclinA*, *Retinoblastoma*, full genes, including the promoter and coding region were achieved with the Triple Master polymerase mix (Eppendorf). A sub-cloning step in the pGEMT vector (Promega) was performed first. The pOtLuc vector was used to fuse the gene in frame with luciferase enabling protein quantification via *in vitro* luciferase assay [18]. The pOtoxLuc vector was designed to facilitate the selection of overexpression/antisense transformants on the basis of luminescence levels produced from luciferase fused to the CCA1 promoter (Figure S3). POtoxLuc allows the expression of the sequences of interest in sense or antisense orientation under control of the strong *High Affinity Phosphate Transporter* promoter (pHAPT). *CyclinA* coding sequence and antisense of 3′-end sequence of Retinoblastoma coding sequence (from position 2964 to 1824) were cloned in pOtoxLuc. Overexpression of CyclinA was confirmed by western blot, and Knock-down of *Retinoblastoma* by quantitative RT-PCR.

### Transformation of *Ostreococcus* tauri and screening of transformants

Transformation was performed as previously described [18]. Briefly, *O. tauri* was harvested by centrifugation (8000 g, 8 min, 10°C) after pluronic addition (0.1% final concentration) and cells were gently resuspended in 1 ml 1 M sorbitol. After one supplemental wash, cells were resuspended in 50 to 80 µL sorbitol (2 to 3×10^10^ cells per ml) and incubated with 5 µL of linearised DNA (1 µg/µL) before electroporation using a Bio-rad Gene Pulser apparatus (field strength 6 kV/cm, resistor 600 Ω, capacitor 25 µF). Cells were transferred into culture medium for 24 h. Stable transformant colonies were selected in semi-solid medium at 0.2% w/v agarose (low melting point agarose, Invitrogen) in Keller Medium supplemented with G418 (Calbiochem) at 1 mg/ml concentration. Individual clones were transferred in liquid medium in 96-well microplate until they reached stationary phase (4 to 6 10^7^ cell/ml). Luciferase reporter lines were selected on the basis of reproducible patterns of luminescence under LD conditions. Overexpressing/Antisense lines were first selected from the lines displaying the highest luminescence level and then analyzed either by quantitative RT-PCR or by western blot.

### RNA extraction and quantitative RT–PCR

RNA was extracted using RNeasy-Plus Mini kit (Qiagen, Hilden, Germany) following the manufacturer's instructions. Contaminating DNA was removed using Q1 RNAse-free DNAse (Promega). Absence of DNA contamination was checked by PCR. Reverse transcription was performed using the PowerScript Reverse Transcriptase synthesis kit (BD Bioscience, Palo Alto, CA). Real-time PCR was carried out on a LightCycler 1.5 (Roche Diagnostic) with LightCycler DNA Master SYBR Green I (Roche Molecular Biochemicals). Primers were designed with LightCycler Probe Design2 software (Roche Diagnostic, Mannhein, Germany). Primers are available in Table S1. Results were analyzed using the comparative critical threshold (ΔΔ*C*T) method. The *O. tauri* elongation factor 1α (EF1α was used as internal reference. The analyses were performed in duplicate. Errors (SD) were usually below 1%.

### Protein extraction and luciferase assay

Proteins were extracted in CCLR buffer (100 mM potassium phosphate pH 7.8, 1 mM EDTA, 1 mM DTT, 1% TritonX-100, 10% glycerol). For affinity purification, protein extracts were diluted 5 time in CCLR with antiprotease without glycerol and further incubated with either p9^CKShs1^ sepharose beads (Corellou, 2005) or specific anti-*Ostreococcus* CyclinA bound to protein A sepharose on a rotator at 4°C for one hour as previously described [40]. After western blotting, protein detection was achieved by enhanced chemiluminescence detection.

Luminescence of translational luciferase fusion proteins (50 µl of protein extract) was recorded on Centro LB 960 luminometer (Berthold Technologies, Germany), 1 minute after injection of 80 µl of luciferase assay reagent buffer (20 mM Tricine pH 7.8, 5 mM MgCl2, 0.1 mM EDTA, 3.3 mM DTT, 270 µM coenzyme A, 500 µM luciferin, 500 µM ATP). The luciferase background was determined by running controls lacking the primary antibody (e.g anti-CyclinA) and substracted for each time point. We also checked using various amount of recombinant luciferase expressed under control of the High affinity phosphate promoter that luciferase is not immunoprecipitated by antibodies or bound to P9. In these control experiments, the luciferase activity was below 0.1% of the initial luciferase activity in the extract before immunoprecipitation.

### Quantitative measurement of cAMP

For each sample, 1 ml of cell culture (corresponding to five millions of cells), were extracted in HHBS buffer. cAMP measurements were performed with cAMP I HitHunter assay kit for cells in suspension (DiscoveRx Corp., CA) according to the manufacturer's instructions. Luminescence was recorded in 96 wells microplate using a Centro LB 960 luminometer (Berthold Technologies, Germany). A cAMP standard curve was established to quantify the cAMP levels which were normalized to the cell number as determined by flow cytometry.

## Supporting Information

Figure S1 Effect of high light intensity on cell division. (A) Cells were synchronized for five days in LD 12, 12 at 35 µmol.quanta.m-2.sec-1 and then exposed to high light (100 µmol quanta.m-2.sec-1) of various durations (from 3 to 12 hours) before being transferred to darkness. Cells in S and G2/M phases were determined by flow cytometry (n = 20,000). (B) Cell number as determined by flow cytometry for each light duration. Note that for 10 or 12 hours of light, more than one division was observed at the cell population level indicating that some cells underwent two divisions in a row.
(3.50 MB TIF)

Figure S2 Effect of light intensity on cAMP level. Cells were entrained for five days in LD 12, 12 and then exposed for 12 hours to light of various intensities from dawn. Levels of cAMP in cell extracts were quantified as described in the Materials and Methods section.
(2.40 MB TIF)

Figure S3 Schematic map of the overexpression/antisense pOtoxLuc vector. Overexpression or knock-down of the gene of interest is achieved by expressing the sequence of interest in sense or antisense orientation under control of the strong High Affinity Phosphate Transporter promoter (pHAPT). The KanMx sequence encoding G418 resistance is driven by the histone H4 promoter (pH 4). The Luciferase (Luc) marker under control of CCA1 promoter (pCCA1) is used to select lines with high level of expression in a primary screening. Sequences of interest are cloned in sense or antisense orientation in the cloning sites (CS).
(1.76 MB TIF)

Figure S4 Quantification of the *Retinoblastoma* transcript in a representative Rb-knock down line. Cells were entrained in LD: 12,12 and then subjected to 8 hours of light at 100 µmol quanta.m^−2.^sec^−1^ from dawn. *Retinoblastoma* mRNA level was monitored by real time quantitative RT-PCR in Rb-Kd and wild-type (WT) cells and normalized to EF1α. Values are mean of duplicates.
(2.00 MB TIF)

Table S1 List of oligonucleotides used for quantitative RT-PCR.
(0.01 MB PDF)
