# Supplementary figures and images for: Integration of Light Signals by the Retinoblastoma Pathway in the Control of S Phase Entry in the Picophytoplanktonic Cell Ostreococcus
Source: PLoS Genet. 2010 May 20;6(5):e1000957. doi: 10.1371/journal.pgen.1000957 (PMC2873908; doi:10.1371/journal.pgen.1000957)

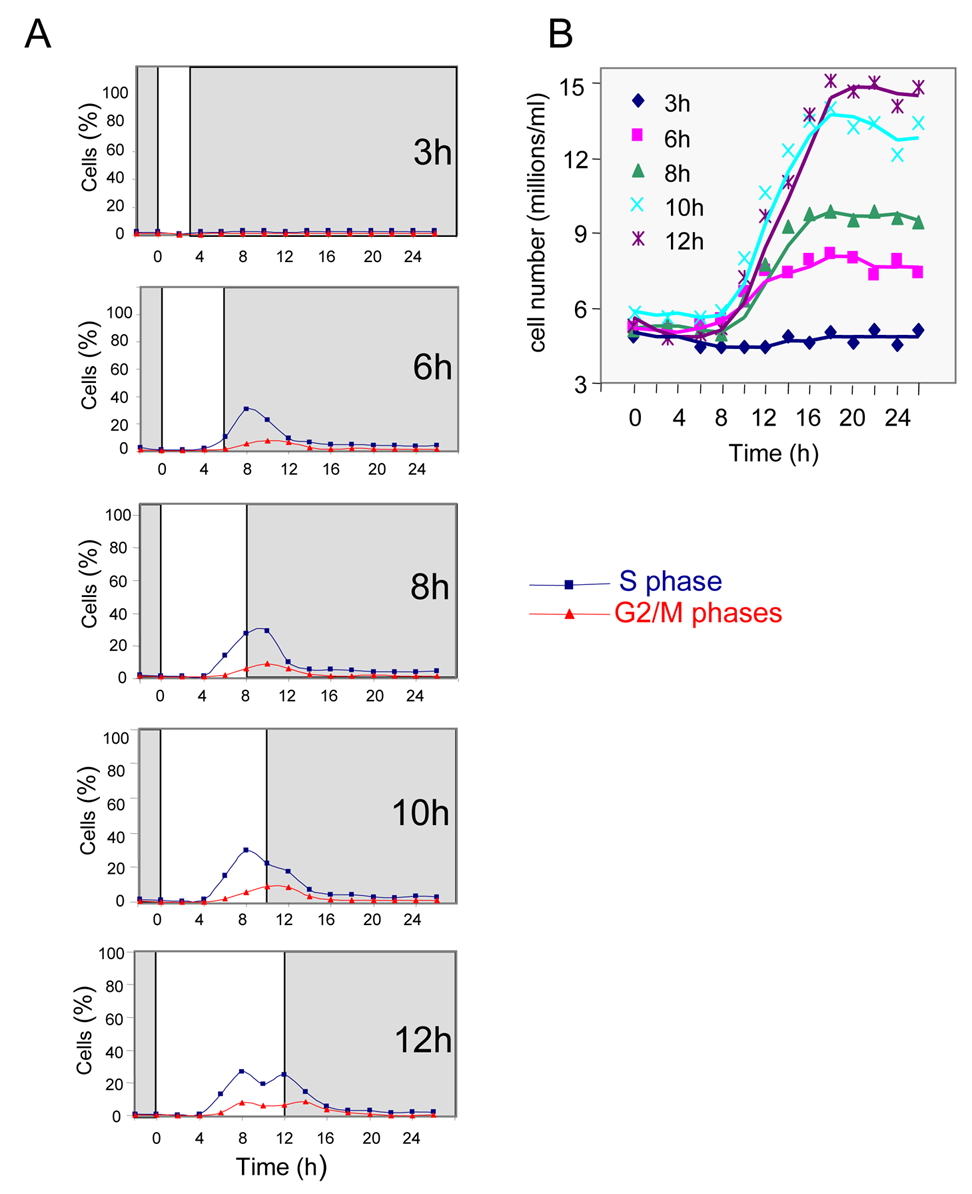

Supplement: Figure S1 — Effect of high light intensity on cell division. (A) Cells were synchronized for five days in LD 12, 12 at 35 µmol.quanta.m-2.sec-1 and then exposed to high light (100 µmol quanta.m-2.sec-1) of various durations (from 3 to 12 hours) before being transferred to darkness. Cells in S and G2/M phases were determined by flow cytometry (n = 20,000). (B) Cell number as determined by flow cytometry for each light duration. Note that for 10 or 12 hours of light, more than one division was observed at the cell population level indicating that some cells underwent two divisions in a row. (3.50 MB TIF) [file pgen.1000957.s001.tif]

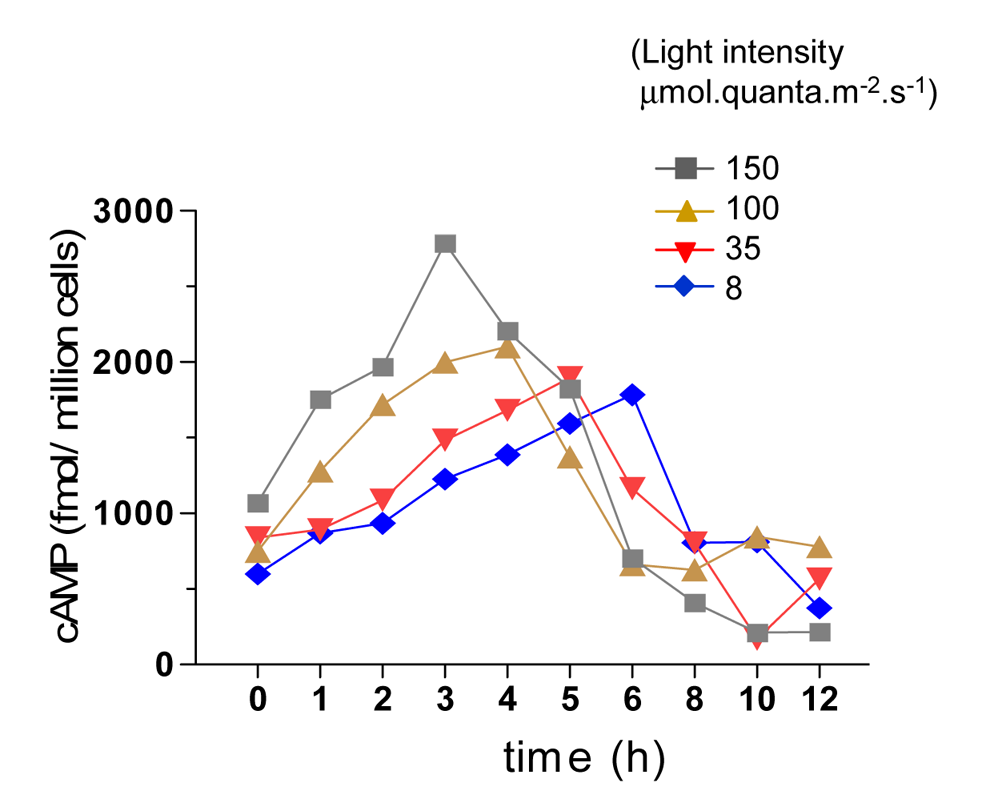

Supplement: Figure S2 — Effect of light intensity on cAMP level. Cells were entrained for five days in LD 12, 12 and then exposed for 12 hours to light of various intensities from dawn. Levels of cAMP in cell extracts were quantified as described in the Materials and Methods section. (2.40 MB TIF) [file pgen.1000957.s002.tif]

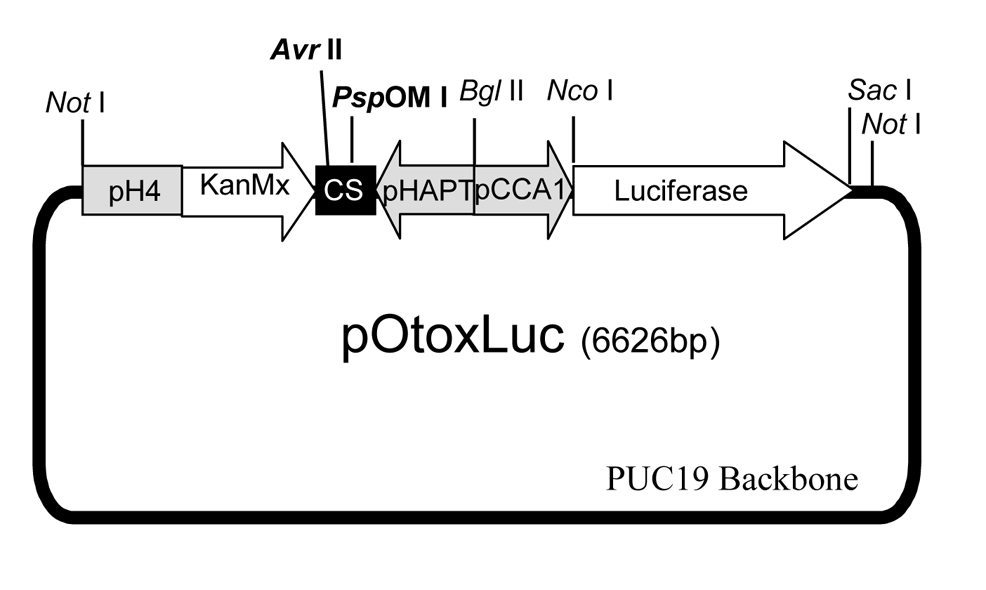

Supplement: Figure S3 — Schematic map of the overexpression/antisense pOtoxLuc vector. Overexpression or knock-down of the gene of interest is achieved by expressing the sequence of interest in sense or antisense orientation under control of the strong High Affinity Phosphate Transporter promoter (pHAPT). The KanMx sequence encoding G418 resistance is driven by the histone H4 promoter (pH 4). The Luciferase (Luc) marker under control of CCA1 promoter (pCCA1) is used to select lines with high level of expression in a primary screening. Sequences of interest are cloned in sense or antisense orientation in the cloning sites (CS). (1.76 MB TIF) [file pgen.1000957.s003.tif]

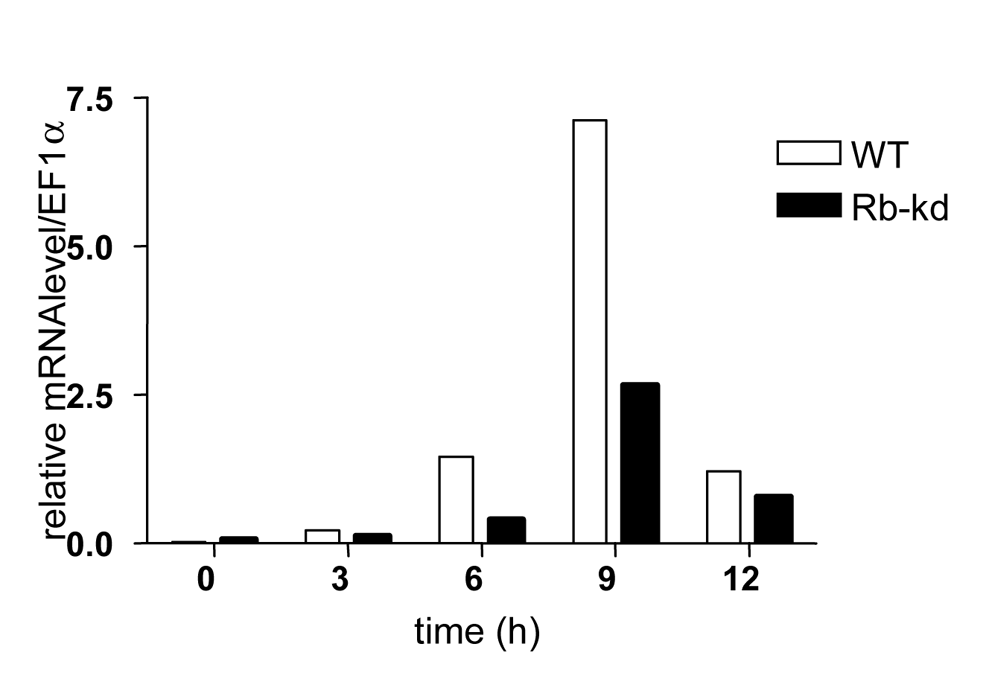

Supplement: Figure S4 — Quantification of the Retinoblastoma transcript in a representative Rb-knock down line. Cells were entrained in LD: 12,12 and then subjected to 8 hours of light at 100 µmol quanta.m−2.sec−1 from dawn. Retinoblastoma mRNA level was monitored by real time quantitative RT-PCR in Rb-Kd and wild-type (WT) cells and normalized to EF1α. Values are mean of duplicates. (2.00 MB TIF) [file pgen.1000957.s004.tif]
