# Supplementary material for: Integration of Light Signals by the Retinoblastoma Pathway in the Control of S Phase Entry in the Picophytoplanktonic Cell Ostreococcus
Source: PLoS Genet. 2010 May 20;6(5):e1000957. doi: 10.1371/journal.pgen.1000957 (PMC2873908; doi:10.1371/journal.pgen.1000957)
Supplement: Table S1 — List of oligonucleotides used for quantitative RT-PCR. (0.01 MB PDF) [file pgen.1000957.s005.pdf]

Supplemental Table 1. List of oligonucleotides used for quantitative RT-PCR

Target gene	Foward primer	Reverse primer	efficiciency
CDKA	TGGTTAATCACGCGCC	TGTGTTCCCTGTAGTCAT	1.89
CDKB	ACTCCGGTCGATATGTG	CTTCCATTGCGGGAAC	1.93
Cyclin A	AAGTGCCTGGAGTTTCT	AGTTGCGACCTCATTGTA	1.9
Cyclin D	CTGCTTGCATATTGCGG	GCTGGTTCAACAGGCTC	1.82
Cyclin B	TCAAACGGGATCGTGG	CTATGGACACGTCGTCT	1.88
Rb (3' UTR)	AACAGACCAATCTGGTG	CTCAGGGTCAGTTTGCTC	1.92
EF1 α	GACGCGACGGTGGATCAA	CGACTGCCATCGTTTTACC	1.99
